# The Role of Rab Proteins in Neuronal Cells and in the Trafficking of Neurotrophin Receptors

**DOI:** 10.3390/membranes4040642

**Published:** 2014-10-06

**Authors:** Cecilia Bucci, Pietro Alifano, Laura Cogli

**Affiliations:** Department of Biological and Environmental Sciences and Technologies (DiSTeBA), University of Salento, Via Provinciale Lecce Monteroni 165, Lecce 73100, Italy; E-Mails: pietro.alifano@unisalento.it (P.A.); lcog@inwind.it (L.C.)

**Keywords:** neurotrophins, neurotrophin receptors, Trk receptors, p75NTR, Rab proteins, axonal transport, neurodegenerative diseases, membrane trafficking, signaling

## Abstract

Neurotrophins are a family of proteins that are important for neuronal development, neuronal survival and neuronal functions. Neurotrophins exert their role by binding to their receptors, the Trk family of receptor tyrosine kinases (TrkA, TrkB, and TrkC) and p75NTR, a member of the tumor necrosis factor (TNF) receptor superfamily. Binding of neurotrophins to receptors triggers a complex series of signal transduction events, which are able to induce neuronal differentiation but are also responsible for neuronal maintenance and neuronal functions. Rab proteins are small GTPases localized to the cytosolic surface of specific intracellular compartments and are involved in controlling vesicular transport. Rab proteins, acting as master regulators of the membrane trafficking network, play a central role in both trafficking and signaling pathways of neurotrophin receptors. Axonal transport represents the Achilles' heel of neurons, due to the long-range distance that molecules, organelles and, in particular, neurotrophin-receptor complexes have to cover. Indeed, alterations of axonal transport and, specifically, of axonal trafficking of neurotrophin receptors are responsible for several human neurodegenerative diseases, such as Huntington’s disease, Alzheimer’s disease, amyotrophic lateral sclerosis and some forms of Charcot-Marie-Tooth disease. In this review, we will discuss the link between Rab proteins and neurotrophin receptor trafficking and their influence on downstream signaling pathways.

## 1. Introduction

Neurotrophins constitute a small family of growth factors that play a key role in several different aspects of the development and maintenance of the nervous system. The first factor that was discovered in 1951 and subsequently purified was the nerve growth factor (NGF), which was initially identified as a secreted factor able to stimulate growth of the sensory and sympathetic neurons of chicken embryos [1,2]. Approximately 30 years later, brain-derived neurotrophic factor (BDNF) was identified, followed by neurotrophin-3 (NT-3) and neurotrophin-4 (NT-4) in the 1990s [3,4,5,6]. Importantly, all neurotrophins are crucial not only for the development of the nervous system, as was initially believed, but also for the adult brain, as they have roles in plasticity, neurodegeneration and neuroprotection, thus reinforcing their relevance for the nervous system [7].

Secreted neurotrophins bind to their receptors and initiate signaling cascades that serve to communicate with the nucleus in order to regulate neuronal growth, differentiation, survival and maintenance [8]. Signaling does not take place exclusively at the plasma membrane but also in signaling compartments along the endocytic route, leading to spatial compartmentalization and specific processing of the signal [9]. Thus, the regulation of trafficking of neurotrophins and of neurotrophin receptors is important to control neuronal functions, and key regulatory factors of trafficking are the Rab proteins [9,10].

Rab proteins are a large family small GTPases that direct intracellular vesicular trafficking. Approximately 70 different Rab proteins are present in mammalian cells. Rab proteins are localized on specific intracellular compartments and are thus often used as markers for organelles (e.g., Rab11 for recycling endosomes, Rab5 for early endosomes, and Rab7 for late endosomes) [10,11]. Rab proteins act as molecular switches, cycling from an inactive GDP-bound state to an active GTP-bound state, and vice versa. In the active state, which is induced by GEFs (guanine nucleotide exchange factors), Rab proteins are able to bind to a number of effector proteins that regulate a wide range of membrane transport processes and cell signaling transduction pathways [12]. After GTP-hydrolysis is stimulated by GAPs (GTPase activating proteins), Rab proteins return to their GDP-bound form [12]. Rab proteins are involved in virtually all steps of vesicular trafficking, such as vesicle formation, vesicle transport along cytoskeletal elements, vesicle tethering and fusion to the target compartments [10,11]. Importantly, Rab GTPases also determine compartment identity and function by ordering membrane trafficking events [13,14]. In addition, a number of Rab proteins directly interact with cargoes, such as signaling receptors, integrins and ion channels, thus directly regulating their fate [15,16].

In this review, we will focus on the role of Rab proteins in the regulation of neurotrophin receptors trafficking, highlighting their role in modulating neurotrophin signaling and functions. Furthermore, we will consider neuropathological conditions caused by defective neurotrophin signaling, possibly due to alterations of neurotrophin axonal transport.

## 2. Neurotrophins and Their Receptors

Growth factors play crucial roles in development and maintenance of the mammalian nervous system. Most of these factors, including fibroblast growth factors (FGF) 1 and 2, insulin-like growth factors (IGF) I and II, gliostatin/platelet-derived endothelial cell growth factor (gliostatin/PD-ECGF), neurturin, netrin, epidermal growth factor (EGF), transforming growth factor-β (TGF-β), and tumor necrosis factor-α (TNF-α), have pleiotropic effects on different systems [17,18]. Other factors exhibit more restricted activity on the nervous system and are therefore classified as “neurotrophic factors.” The most intensively studied neurotrophic factors include ciliary neurotrophic factor (CNTF), glial-derived neurotrophic factor (GDNF), and the neurotrophic factors belonging to the nerve growth factor (NGF) family, known as “neurotrophins” [19,20]. Although this term is sometimes used as a synonym for neurotrophic factor, it is more properly reserved for this small family of structurally related factors that secrete into the nervous system and have a coherent signaling mechanism involving two types of receptors [21].

### 2.1. Neurotrophins

Neurotrophins, comprising mammalian nerve growth factor (NGF), brain derived neurotrophic factor (BDNF), neurotrophin-3 (NT-3) and neurotrophin-4/5 (NT-4/5), are a small, well-characterized family of secreted growth factors [21]. They share approximately 50% amino acid homology and play critical roles in different aspects of the development and maintenance of the nervous system, such as neuronal migration and differentiation, myelination, neurite outgrowth, axonal elongation, synaptic modulation and apoptosis [21]. They are generated in the rough endoplasmic reticulum as pro-neurotrophin precursors of approximately 240–260 amino acids and are further processed by proteases to be secreted as homodimeric proteins in the extracellular space (monomer length: 118–129 aa) [22]. Once synthesized, neurotrophins can be sorted into the constitutive or regulated secretory pathway, and sorting seems to be regulated by the efficiency of protease processing [22]. Interestingly, pro-neurotrophins as well as other peptides are biologically active and liberated by the cleavage of pro-neurotrophins, indicating that a tight regulation of cleavage is important for neuronal functions and survival [23,24,25]. Importantly, in the nervous system, neurotrophin secretion increases after injury, thus indicating a key role of neurotrophins in axonal regeneration [26].

### 2.2. Neurotrophin Receptors

**Figure 1:**
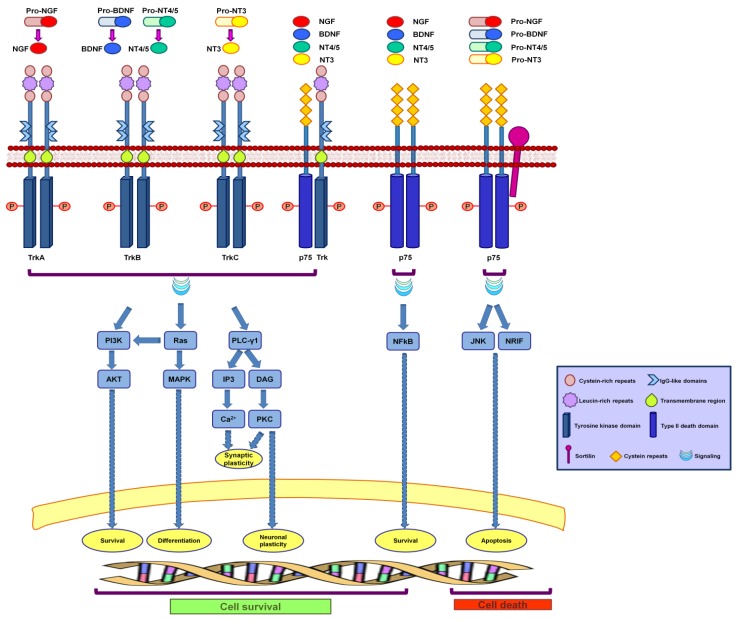
Signal transduction pathways activated by neurotrophins. Mature neurotrophins bind to their preferred Trk receptors (TrkA for NGF, TrkB for BDNF and NT4/5 and TrkC for NT3) and induce receptor dimerization and phosphorylation at specific tyrosine residues. The phosphorylation of Trk receptors activates three major signaling pathways: the PI3K, Ras and PLC-γ1 pathways. PI3K, via AKT kinase, promotes neuronal survival; Ras activates PI3K and MAPK and induces neuronal differentiation; PLC-γ1 leads to production of IP3 and DAG, the first promoting calcium release from internal stores, which is important for synaptic plasticity, and the second carrying out PKC activation, thus inducing synaptic and neuronal structural plasticity. All of these pathways are also activated by binding of neurotrophins to heterodimers formed by Trk and p75NTR. Cell survival is also promoted by the activation of NFkB induced by the binding of neurotrophin to p75NTR, while apoptosis is induced by neurotrophins binding to the p75NTR-sortilin complex, which activate JNK and NRIF.

To accomplish their role in the nervous system, neurotrophins recognize and activate two different classes of transmembrane receptor proteins: tropomyosin-receptor kinases (Trks) and the neurotrophin receptor p75 (p75NTR) (Figure 1).

Trks are transmembrane glycoproteins of approximately 140 kDa. Trk proteins have four domains: an intracellular tyrosine kinase domain, a single transmembrane region, an extracellular neurotrophin-binding domain consisting of two cysteine-rich regions separated by a leucine-rich repeat, and two IgG-like domains near the plasma membrane. Neurotrophins bind to the second IgG-like domain, induce receptor dimerization and trigger tyrosine kinase activity by phosphorylation [26]. The three most studied Trks are TrkA, TrkB and TrkC. Each neurotrophin preferentially binds to a specific Trk: NGF activates TrkA, BDNF and NT-4/5 activate TrkB while NT-3 binds preferentially to TrkC [27]. In addition, the interaction of neurotrophins with their receptors might be influenced by the splicing variants of Trk receptors. In fact, receptor molecules with deletions in the extracellular domains or in the intracellular kinase domain have been identified [26,28,29]. Specifically, a TrkA splice variant with an 18 bp deletion in the extracellular domain shows decreased activation by NT-3, and a TrkB molecule lacking exon 9 (comprising 33 bp, which encodes the juxtamembrane domain) interacts poorly with NT-4/5 and NT-3, while NGF binding is unaffected compared to full-length receptor [28,29]. Furthermore, truncated forms of the TrkB and TrkC receptors, which lack the tyrosine kinase domain, are unable to dimerize and are thus considered to be dominant negative modulators of Trk signaling, in contrast with their full-length counterparts [30].

p75NTR, a transmembrane glycoprotein receptor of approximately 75 kDa, is a member of the tumor necrosis factor (TNF) receptor superfamily. Unlike the Trk receptors, p75NTR binds to all neurotrophins with approximately similar low affinity and to all pro-neurotrophins with high affinity [25,27]. In the extracellular domain of p75NTR, the four cysteine repeats participate in binding to NGF, while in the intracellular domain, there is a type II death domain similar to those present in other members of the TNF family [25,26,27]. In addition to neurotrophins, several other ligands bind to p75NTR, thus influencing neuronal functions and survival. For example, the soluble β-amyloid precursor protein alpha promotes neurite outgrowth through binding to p75NTR, while β-amyloid peptide and prion peptide 106–126 bind to p75NTR and induce neuronal death [31,32,33]. Notably, β-amyloid peptide also binds to TrkA, and it has been demonstrated that it regulates endocytosis of p75NTR and Trk receptors, thus influencing neuronal survival and differentiation [34]. p75NTR also interacts with a number of other receptors, and these interactions often modulate ligand binding as well as signaling and trafficking. For example, the affinity of p75NTR for pro-neurotrophins increases in the presence of the neuronal type I receptor sortilin [35]. p75NTR is also frequently co-expressed and associated with Trk receptors and increases Trk’s affinity for neurotrophins while reducing ligand-induced Trk receptor ubiquitination, thus delaying Trk receptor internalization and degradation [36,37]. Furthermore, it has been demonstrated that the intracellular domain of p75NTR, generated by neurotrophin-induced receptor cleavage, increases Trk signaling and that activation of p75NTR by pro-NGF is able to suppress TRK-mediated signaling for neuronal survival [38,39]. The role of p75NTR is dual, as it is able to trigger both cellular survival and pro-apoptotic signaling. In physiological conditions, binding of neurotrophin to Trk and p75NTR receptors leads to neuronal survival because the survival signal mediated by Trk suppresses the pro-apoptotic signal from p75NTR [40]. In pathological conditions, this pro-apoptotic signal prevails due to up-regulation of p75NTR or to an increase in pro-neurotrophin concentration [25]. Moreover, an alternative cell survival pathway is promoted by activation of NFkB, which is induced by binding of neurotrophin to p75NTR. In contrast, when pro-neurotrophins bind to the p75-sortilin complex, JNK and NRIF trigger cell death signaling through apoptosis [41].

## 3. Neurotrophin Signaling

### 3.1. Neurotrophin Receptor Signaling

When neurotrophins bind to Trk receptors, they lead to Trk dimerization and phosphorylation of specific tyrosine residues in the cytoplasmic domain, initiating signaling cascades. These phosphorylated tyrosine residues act as docking sites for adaptor proteins that propagate neurotrophin signals [42,43]. The Trk receptors can activate three major pathways: Ras, phosphatidylinositol 3-kinase (PI3K) and phospholipase C-γ1 (PLC-γ1) signal transduction pathways [27,44] (Figure 1). These three signal transduction pathways are also activated by the binding of mature neurotrophins to heterodimers formed by one monomer of Trk and one of p75NTR [41,45,46,47] (Figure 1).

Ras activation is required for neuronal and PC12 cell differentiation [48,49]. Phosphorylation on the Y490 tyrosine residue, located in the Trk juxtamembrane domain, causes the recruitment and phosphorylation of the adapter protein Shc. Phospho-Shc binds to the Grb2-SOS complex, which mediates Ras activation by inducing GDP to GTP exchange, causing many downstream effects, such as the transient stimulation of the PI3K and MAPK pathways [27]. MAPK translocates to the nucleus, where it phosphorylates CREB (cAMP Responsive Element Binding protein), a transcription factor that promotes neuronal cell differentiation [50].

PI3K is activated by an adaptor protein complex, Shc-Gr2-Gab1 [51], or by Ras. In both cases, PI3K produces 3-phosphoinositides that trigger the activation of Akt, a serine-threonine kinase [52]. Akt phosphorylates various proteins that are important in the control of cell survival [53]. For example, Akt phosphorylates and inactivates BAD, a pro-apoptotic Bcl-2 family member [54], preventing the activation of several genes whose products promote apoptosis [55]. Thus, activation of PI3K promotes the survival and growth of neurons [56].

Activation of PLC-γ1 through Trk-mediated phosphorylation causes phosphatidylinositol 4,5-biphosphate hydrolysis in diacylglycerol (DAG) and inositol trisphosphate (IP_3_). The formation of DAG triggers the activity of DAG-regulated isoforms of protein kinase C, whereas IP_3_ leads to the release of Ca^2+^ from cytoplasmic stores, activating Ca^2+^-calmodulin-dependent protein kinases. Both pathways are important for synaptic plasticity [57]. Moreover, it has been demonstrated that the PLC-γ1 pathway modulates transcription and thus protein expression. For example, PLC-γ1 influences expression of peripherin, a 57 KDa type III intermediate filament protein, found primarily in peripheral neurons [58]. Because modulation of peripherin expression has been observed in neuronal degeneration and axonal regeneration [59,60], the PLC-γ1 pathway is believed to have a key role in these processes. Interestingly, we have recently identified peripherin as a Rab7-interacting protein [61], thus suggesting that Rab7 can be directly or indirectly involved in the PLC-γ1 signaling pathway. Importantly, post-translational modification of these receptors is believed to be important for receptor localization and activity. For example, TrkA glycosylation prevents ligand independent activation and it is fundamental for plasma membrane localization of TrkA, for activation of the Ras/MAPK pathway and subsequent induction of neuronal differentiation in PC12 cells [62].

### 3.2. Neurotrophin Receptor Trafficking and the “Signaling Endosome” Hypothesis

Ligand-receptor complexes are internalized by different endocytic pathways and, after internalization, these complexes are mainly delivered to early sorting endosomes. From the sorting endosomes, some receptors (such as transferrin receptors, TfR) are recycled back to the plasma membrane directly or via recycling endosomes, while others (such as epidermal growth factor receptors, EGFR) are sorted, together with their ligands, along the endocytic pathway for degradation in late endosomes and lysosomes. Internalization of ligand/receptor signaling complexes by endocytosis was considered for a long time to be important solely to terminate signaling, which was generally believed to occur only at the plasma membrane. However, it is now clear that there is a high degree of compartmentalization of signaling in order to control and modulate the cell response. Indeed, spatial compartmentalization of signaling is a general mechanism to ensure specific signal processing [63,64]. Furthermore, the “signaling endosome” hypothesis was formulated for neurotrophin signal transduction, which states that after internalization into endosomes, neurotrophins remain attached to their activated receptors and, from the endosomal network, these complexes continue to transmit signals to control essential responses [9]. This hypothesis was formulated on the finding that NGF causes accumulation of activated TrkA receptor bound to NGF, PLC-γ1 and components of MAPK and PI3K signaling pathways in endosomes of PC12 cells and nociceptive neurons [65,66]. For example, it was initially demonstrated that, after endocytosis, NGF/TrkA complexes keep signaling in uncoated endocytic vesicles [65,67]. Furthermore, after NGF binds to TrkA and p75NTR, both receptors are internalized in early sorting endosomes. However, while p75NTR is recycled to the plasma membrane, TrkA moves to late endosomes and lysosomes [68]. Interestingly, activated TrkA receptors are found not only in early endosomes but also in late endosomes, suggesting that these organelles could also represent signaling platforms for neurotrophin receptors [68]. Notably, Trk and p75NTR receptors regulate each other, thus influencing signaling and cell responses. Indeed, disruption of p75NTR recycling results in reduced activation of TrkA, indicating a role of p75NTR recycling for TrkA activation and displaying the importance of trafficking in the signaling of neurotrophin receptors [68]. Additionally, upon NGF stimulation, TrkA regulates the intracellular localization of p75NTR, affecting localization of signaling molecules and consequently driving the activation of specific signal transduction pathways [69]. Moreover, pro-neurotrophins do not directly activate Trk receptors, but their endocytosis and cleavage are able to induce Trk activation [70].

Interestingly, different biological responses to NGF are controlled by signaling from different intracellular locations. For example, neuronal differentiation is promoted by active Trks localized to endosomes, whereas survival is accomplished by Trk signaling at the cell surface [71]. Furthermore, NGF and NT-3 bind and signal through TrkA, but only NGF supports survival because it induces internalization and retrograde axonal transport of TrkA [72].

Neurotrophin-TrkA receptor complexes enter the cell by clathrin-mediated endocytosis, as internalization is blocked by monodansylcadaverine, a drug that inhibits the clustering and internalization of ligand-receptor complexes into clathrin-coated vesicles [73]. Moreover, the activated TrkA receptor is located along with components of the ERK1/2 signaling pathway [74] and is connected with PI3-K pathway [73]. In contrast, neurotrophin-p75NTR receptor complexes also use lipid raft mediated internalization [75]. Interestingly, NGF binding induces TrkA to translocate and concentrate in cholesterol-rich microdomains, or lipid rafts, a step that is fundamental for efficient activation of ERK signaling before internalization into endosomes [76]. Trk receptors are also internalized by macropinocytosis, which requires Rac and Pincher (PINocytic CHapERon), a trafficking protein [77]. Macropinocytosis, which is triggered by clathrin-mediated endocytosis, is able to generate long-lived TrkA-signaling endosomes, also known as NGF signalosomes, which are connected to the downstream activation of ERK5 but not ERK1/2, [78,79], eliciting long-term activation that promotes neuronal differentiation and survival [78,79]. It has been questioned why Trk receptors are able to promote neuronal differentiation and survival whereas other tyrosine-kinase receptors, such as EGFR, exert poor neuromodulatory functions. Comparison of Trk- and EGFR-containing endosomes revealed that they are formed and processed by distinct mechanisms. Indeed, both TrkA and EGF are retrogradely transported from the axon to the soma via multivesicular bodies, but the TrkA multivesicular bodies are resistant to signal termination [80]. Notably, NGF induces TrkA ubiquitination, and ubiquitinated receptors interact with the proteasome to become deubiquitinated before delivery to lysosomes for degradation [81]. Regulation of TrkA stability by the ubiquitin-proteasome potentiates neurite outgrowth [82,83].

It is interesting to note that neurotrophins are able to regulate endocytosis. Indeed, NGF signaling through TrkA increases clathrin–mediated endocytosis, thus contributing to trophic actions [84], while activated TrkA in a vesicle is able to facilitate intracellular transport of that vesicle by increasing movement and velocity [85]. Furthermore, the cell distribution of Trk receptors is controlled by transcytosis, as receptors on cellular soma and dendrites are endocytosed and transported via recycling endosomes into axons [86].

## 4. Neurotrophin Receptor Trafficking: The Role of Rab Proteins

### 4.1. Neuronal Trafficking and Axonal Transport

The intracellular transport of molecules and vesicles is of vital importance for the life of a cell. Neurons are highly polarized cells with long axons and dendrites. Therefore, perhaps their most difficult task is to maintain an efficient transport of molecules over long distances, as transport from and to the cell periphery has to cover much greater distances than in other cell types. While the majority of cells are approximately tens of micrometers long, neurons have long dendrites and much longer axons that can reach the length of 1 meter in the peripheral nervous system. Communication between dendrites and axons and the cellular soma is important not only for neuronal functions, but, even more importantly, for neuronal survival. Axonal transport allows synapses to send signals to the soma and vice versa. Additionally, proteins synthesized in the cell soma require an efficient transport mechanism in order to reach the axon and dendrites. Furthermore, there are some mRNAs that have to be transported to dendrites and/or axons in order to be translated locally [87]. For example, axonal injury activates local protein synthesis, thus indicating that axonal regeneration is dependent on RNA axonal transport [87]. In addition, signals from the tips of the axons have to reach the cytoplasm and/or the nucleus in order to elicit cell responses. Thus, the considerable distance between the tip of the axon, where neurotrophins bind to their receptors, and the soma, where the signaling molecules have to arrive in order to initiate a specific gene expression response, requires an efficient and active axonal transport of molecules and organelles, leading to multiple cycles of phosphorylation/dephosphorylation on endosomes, thus regenerating signals [8,9]. Anterograde axonal transport is required to transfer molecules and newly formed organelles towards the synapses, while retrograde axonal transport is important for delivery of molecules and organelles to the soma [88,89].

Experiments on axonal transport kinetics allowed for the identification of slow and fast axonal transport. Cytosolic and cytoskeletal proteins are transported by slow axonal transport with kinetics of approximately 0.2–5 mm/day (0.01–0.001 micron/Sec), whereas fast axonal transport moves membranous organelles at a rate of 100–400 mm/day (1–5 microns/Sec) [90].

The axonal transport machinery consists mainly of two components: molecular motors and cytoskeletal elements, on which the motors move. Long-range axonal transport requires an intact tubulin cytoskeleton with kinesins being mainly anterograde motors while dynein is responsible for retrograde axonal transport towards minus-ends of microtubules. The most important kinesin for axonal transport is kinesin-1, a heterotetramer consisting of two catalytic kinesin heavy chains and two accessory kinesin light chains [91]. Kinesin-1 is important to transport trophic factor, receptors, ionic channels, organelles, precursors of synaptic vesicles and even dynein towards axonal terminals [92]. The active retrograde transport of cargoes from synaptic terminals to the cell soma is mediated by the dynein-dynactin system [89]. Dynein is a complex of two heavy chains, two intermediate chains, three intermediate light chains and six light chains, while dynactin is a dynein activator that binds to microtubules, enhancing dynein motor efficiency and mediating the interaction with transporter molecules [93,94,95].

The actin cytoskeleton and its myosin motors also have an important role in axonal transport. First, myosin V proteins are highly expressed in the brain and are important for targeting synaptic vesicles, mitochondria, neurofilaments and RNAs to the axon [96]. For example, neurofilament transport is dependent on actin and myosin [97], and a lack of myosin Va causes an increased duration of transport pauses, leading to a reduction in the efficiency of slow anterograde axonal transport of neurofilaments [98]. In *Drosophila melanogaster* neurons, myosin V and myosin VI depletion causes an increase of mitochondrial mean velocity, suggesting that, by opposing to protracted microtubule-based movements, they possibly facilitate organelle docking [99]. Furthermore, myosin VI is important for the localization of axonal proteins, as disruption of myosin VI leads to dendritic localization of axonal proteins, possibly by an increase of endocytosis at dendrites that stimulates transcytosis [100]. Importantly, absence of myosin Va in humans causes Griscelli syndrome, a neurological disease [101]. Fast retrograde transport in motor neurons also requires actin microfilaments [102,103]. Indeed, expression of a dominant negative form of myosin Va causes a general reduction of movement in dendrites and axon [103]. In axons, in particular, retrograde speed of large dense core vesicles is significantly reduced, suggesting that myosin Va facilitates retrograde axonal transport [103]. Importantly, tetanus toxin-containing retrograde carriers colocalize with myosin Va and depletion of myosin Va causes slower retrograde axonal transport, suggesting a requirement for a coordination between myosin and microtubule-dependent motors [102].

### 4.2. Rab Proteins and Neuronal Trafficking

Rab proteins coordinate vesicular trafficking, regulating the different aspects of vesicle formation, movement, targeting and fusion [10,11,13,14]. They are recruited on the forming vesicles and control formation of the vesicle from a donor compartment, selection of the cargo of the vesicle, transport of the vesicle on cytoskeletal tracks to reach the target compartment and tethering and fusion of the vesicle with the target compartment [10,13,15]. Each Rab protein, in order to accomplish all of these functions, interacts with several molecular partners and recruits them on the vesicle membrane [10,11,12,13,15,16]. There are approximately 70 different Rab proteins in mammalian cells, and this complexity is required to regulate all different transport steps [10,11,12,13,15,16]. In specialized cells, either specific Rab proteins are present in order to control particular transport routes or a given Rab protein is able to accomplish additional specific functions by, for example, interacting with cell-specific effector proteins. A number of Rab proteins are specifically and/or predominantly expressed in the brain. Here we will analyze Rab proteins that regulate specific transport pathways present in neurons or specific neuronal functions and then, specifically, we will focus on Rab proteins that control neurotrophin trafficking and signaling.

The Rab3 subfamily, composed of Rab3A, Rab3B, Rab3C and Rab3D, is the first group of Rab proteins that has been associated with neuronal-specific trafficking. Indeed, the Rab3 subfamily is responsible for the docking, fusion and recycling of synaptic vesicles and, thus, for regulated secretion and neurotransmitter release [104,105]. Rab3A, in particular, is required for the assembly and transport of vesicles in fast anterograde axonal transport [106,107]. The ligation of the sciatic nerve causes accumulation of Rab3A-positive vesicles proximal of the ligation site, but no retrograde accumulation is observed [107]. Furthermore, presynaptic vesicles containing APP are Rab3a-positive, and in Rab3aGAP knock-down brains, a reduction of kinesin-1 and two Rab3a-interacting proteins is observed, indicating that Rab3a GTPase activity is required for correct assembly of APP-containing vesicles [106].

Rab8 regulates trafficking of TGN to the plasma membrane, which is important in neurons, as depletion of Rab8 prevents neurite outgrowth and inhibits anterograde formation and transport of vesicles [108]. Indeed, it has been demonstrated that Rab8 antisense oligonucleotides inhibit movement of anterograde vesicles and growth cone activity [108]. Furthermore, in neuronal cells treated with Rab8 antisense oligonucleotides and labeled with Bodipy-ceramide, fluorescence was restricted to the Golgi and no labeled post-Golgi vesicles were detected, indicating that Rab8 is fundamental for the formation and/or budding of vesicles from the Golgi [108]. Furthermore, Rab8 also localizes to recycling tubules that fuse with the plasma membrane, delivering membranes for protrusion formation, thus explaining its role in neurite outgrowth [109].

Another Rab protein with a specific role in neurons is Rab13. Although Rab13 regulates transport of TGN to recycling endosomes, it is also important for the regulation of neurite outgrowth, possibly because of the importance of supplying membranes to recycling endosomes in order to deliver membranes to the cell surface in order to support neurite outgrowth [110].

### 4.3. Rab Proteins and Anterograde Axonal Transport

Anterograde transport of molecules released into the axonal termini is either a mechanism of neurotrophic support or a quick way to respond to the physiological demand of molecules or membranes. For example, the anterograde transport of plasmalemmal precursor vesicles, which are found in growing neurites and involved in plasma membrane expansion, is mediated by a complex formed of three elements, c-Jun N-terminal kinase-interacting protein 1 (JIP1), kinesin-1 light chain (KLC) and GTP-bound Rab10 [111,112,113]. The biogenesis of Rab10-positive vesicles, necessary for axonal outgrowth, is also controlled by Myosin Vb, indicating cooperation between actin cytoskeleton, tubulin cytoskeleton and motor elements, in order to achieve efficient axonal transport [114]. Rab10 regulates trafficking of TGN to the plasma membrane, and it has been demonstrated that expression of wildtype or constitutively active Rab10 induces axonal arborization, while expression of a dominant negative form of Rab10 inhibits it [115,116]. Indeed, activation of Rab10 stimulates trafficking and fusion of membrane precursor vesicles with the plasma membrane, thus supporting axonal growth [116].

### 4.4. Rab Proteins and Anterograde Trafficking of Neurotrophin Receptors

**Figure 2:**
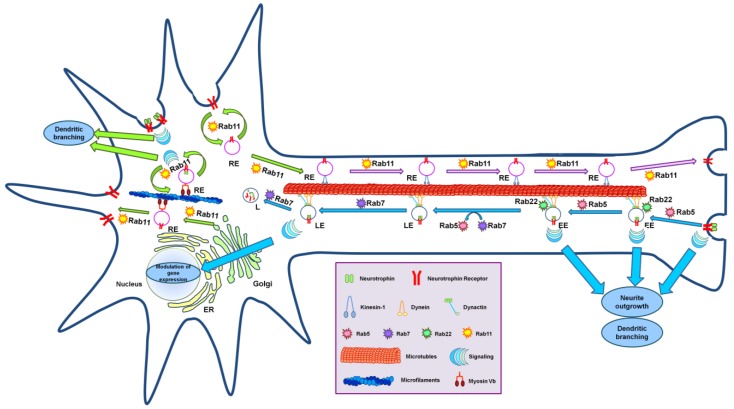
Regulation of neurotrophin receptor trafficking by Rab GTPases. Neurotrophins bind to their receptors at the axonal terminal. After binding, receptors are activated and the activated complexes are internalized into Rab5-, Rab21- and Rab22-positive early endosomes (EEs), which are also called signaling endosomes because, in these vesicles, signaling continues and is able to activate cascade pathways that promote neurite outgrowth and dendritic branching. Signaling endosomes undergo a Rab conversion mechanism (Rab5 is replaced by Rab7) and are retrogradely transported along the axon toward the cell body to Rab7-positive late endosomes (LEs). This retrograde transport on microtubules is mediated by the dynein-dynactin motor complex. LEs are also signaling organelles that promote modulation of gene expression in the nucleus. Neurotrophin receptors after synthesis are inserted into the plasma membrane of dendrites and the cell body, where they can bind the ligand and induce dendritic branching. In the absence of neurotrophins, receptors are transcytosed via Rab11-positive recycling endosomes (REs) and reach the axonal termini via anterograde transport mediated by kinesin-1 on microtubule rails.

After synthesis in the endoplasmic reticulum (ER) and maturation in the ER and the Golgi, vesicles containing Trk receptors are either delivered to the plasma membrane of the soma and dendrites or move towards axon terminals using kinesin motors along microtubule rails [89,117,118]. In particular, it has been demonstrated that TrkB-containing vesicles are anterogradely transported in axons through direct interaction of TrkB with the Slp1-Rab27 complex [119]. Two Rab27 isoforms are expressed in mammalian neurons: Rab27A, which is weakly expressed, and Rab27B, which is strongly expressed [120]. Both of these isoforms are able to associate with the TrkB receptor and regulate the anterograde transport of TrkB-containing vesicles [119]. Notably, sortilin enhances anterograde transport of Trk receptors [118] and, as Rab7b regulates sortilin trafficking in HeLa cells, it would be interesting to test if this GTPase has a role in trafficking Trks in neurons through its interaction with sortilin [121,122]. Direct axonal anterograde transport, however, is not the only route for delivery of Trk receptors to axons. In fact, in sympathetic neurons, an alternative transport route based on transcytosis has been demonstrated. In this endocytosis-dependent pathway, Trk receptors are delivered to somatodendritic surfaces and then endocytosed and transported anterogradely via recycling endosomes into the axon terminal [86]. Transport of neurotrophin receptors into axons by transcytosis occurs via Rab11-positive recycling endosomes and represent a rapid way to mobilize ready-synthesized receptors present on the soma and/or dendritic membranes, thus enhancing neuronal sensitivity [86] (Figure 2). Rab11 regulates recycling endosomes towards the plasma membrane and several studies have shown that Rab11 promotes neuritogenesis through its effector protrudin in hippocampal neurons in culture, in PC12 cells and in dorsal root ganglia [123,124,125]. In addition, the BDNF-TrkB complex is transported to dendrites in Rab11-positive vesicles, which induces dendritic branching [126].

A recent interesting work demonstrated that Rab11-dependent recycling is important to modulate TrkB-regulated synaptic plasticity [127]. In fact, chemical long-term potentiation stimuli increases endocytic recycling of BDNF/TrkB by promoting Rab11 nucleotide exchange [127]. As a result, an increase of BDNF-induced kinase activation is detected and leads to enhanced rat hippocampal neuron survival, thus indicating that recycling endosomes could represent a reserve pool of TrkB functional for long-term potentiation maintenance [127].

### 4.5. Rab Proteins and Retrograde Axonal Transport of Neurotrophin Receptors

Several studies indicate that administration of neurotrophins to the axon terminal causes the formation of a ligand-activated receptor that is endocytosed and enclosed in vesicles that are actively transported along microtubules [128,129]. Thus, neurotrophins, produced and secreted by the target tissue, bind to their specific receptors at the axon terminals and, subsequently, activated neurotrophin/receptor complexes have to be retrogradely transported towards the soma by the microtubule-dependent motor protein dynein in order to modulate gene expression to promote survival of target neurons [130,131]. Key players of retrograde axonal transport of neurotrophin receptors are the Rab5 and Rab7 GTPases and dynein. Rab5 regulates early endosome (EE) formation and fusion along the endocytic pathway while Rab7 regulates transport to late endocytic compartments [132,133,134]. It was demonstrated that the amount of TrkA increases in Rab5- and EEA1-positive vesicles in PC12 cells treated with NGF and that NGF-TrkA complexes are present in early endosomes positive for Rab5 in DRG neurons [66,135]. Therefore, after internalization by clathrin-mediated endocytosis or macropinocytosis, neurotrophin receptor complexes move into Rab5-positive early endosomes [8] (Figure 2). These endosomes then undergo a Rab5-to-Rab7 transition that, in the case of neurotrophin receptor-containing endosomes, is not accompanied by acidification [130,136]. Indeed, it has been demonstrated that p75NTR, TrkB and BDNF share the same retrograde transport pathway that is controlled by the Rab5 and Rab7 GTPases [130]. Docking and fusion of early endosomes require Rab5 and PI3K activity [132]. Rab5 interacts with and transiently recruits the PI3K hVPS34 onto the endosomal membrane in order to obtain, in a confined region of the endosomal membrane, a production of PtdIns(3)P [137]. EEA1, a tethering and fusion factor that interacts with Rab5, binds PtdIns(3)P through a FYVE finger motif and, therefore, Rab5 is able to recruit EEA1 on endosomal membranes by direct interaction but also, more efficiently, on endosomal membrane microdomains enriched with PtdIns(3)P [137].

Rab5-positive endosomes fuse with each other as part of the early endosomal network in order to concentrate cargoes and subsequently progress to late endosomes [138]. TrkA-containing endosomes have lower Rab5 activity in order to divert from the early endosomal network and to become specialized signaling endosomes [139,140]. In fact, in PC12 cells, NGF-activated TrkA decreases Rab5 activity via RabGAP5, a protein that stimulates GTP hydrolysis, and promotes neurite outgrowth, dendritic branching and differentiation [139,140]. In contrast, expression of a constitutively active Rab5 mutant or of Rabex-5 (a specific Rab5 GEF that activates Rab5) inhibits neurite outgrowth, while expression of a dominant-negative Rab5 mutant induces outgrowth [139]. Rabex-5 acts not only on Rab5 but also on Rab21 and Rab22, two Rab proteins localized on early endosomes that regulate endosomal trafficking [141,142]. Rab22 in particular has also been demonstrated to control the sorting of transferrin to recycling endosome ligands [143,144]. Similarly to Rab5, expression of a constitutively active mutant of Rab21 inhibits neurite outgrowth [145,146]. Unlike Rab5 and Rab21, whose expression blocks NGF-signaling endosomes and reduces neurite outgrowth [139,147,148], Rab22 acts as a positive modulator. In fact, its expression promotes the formation of pTrkA-containing signaling endosomes and NGF-induced neurite outgrowth [148].

During endosome maturation from early to late endosome, there is a Rab conversion mechanism in which Rab5 is replaced by Rab7 [138]. Rab7 is a ubiquitous small GTPase that controls transport to late endocytic compartments and regulates the maturation of autophagic vacuoles and of phagolysosomes [133,134,149,150,151]. Endosomes containing Trk receptors also undergo Rab conversion (Figure 2). Interestingly, in NGF-stimulated PC12 cells, expression of a dominant-negative Rab7 mutant causes TrkA storage in endosomes [152]. In addition, inhibition of Rab7 increases TrkA phosphorylation and consequently results in potentiated ERK1/2 phosphorylation, potentiated neurite outgrowth and upregulation of the neuronal differentiation marker GAP-43 (growth-associated protein 43) [152,153]. Therefore, it was hypothesized that Rab7 is able to control the residence time of TrkA in signaling endosomes, holding neurotrophin receptors in endosomes and building a signaling platforms [152,154].

Retrograde axonal transport of signaling endosomes is also controlled by activated kinases. In rat embryonic neurons and PC12 cells, Trk activation of the ERK1/ERK2 kinase pathway causes phosphorylation of the dynein intermediate chain and induces recruitment of cytoplasmic dynein to signaling endosomes, thus stimulating their retrograde transport from axonal periphery to the cell center [155]. Indeed, inhibition of ERK1/ERK2 reduces phosphorylation of the intermediate chain of dynein and, consequently, the amount of dynein present on Trk- and Rab7-containing endosomes [155]. As a consequence, a reduction of motility of Trk- and Rab7-containing vesicles is observed upon inhibition of ERK1/ERK2 [155].

Another important factor for the success of axonal retrograde transport of endosomes is the actin cytoskeleton [156]. In particular, actin depolymerization is fundamental for the initiation of retrograde transport of NGF-TrkA endosomes along the axon. After the formation of Rab5-positive early endosomes, actin depolymerization, mediated by cofilin (an actin filament severing protein) and Rac1 (a regulator of actin cytoskeleton), takes place on NGF-TrkA endosomes, allowing them to start their journey to the soma. These actin-regulatory endosomal components are not recruited on NT3-TrkA endosomes, resulting in an inability of NT3 to elicit TrkA signaling endosomes, which is most likely due to the instability of NT3-TrkA complexes in the acidic endosomal environment [156]. Indeed, axonal retrograde transport of NT3-TrkA-containing endosomes occurs only upon treatment with bafilomycin, a drug that blocks vacuolar ATPase and inhibits endosomal acidification [156].

In neurons, autophagosomes also undergo retrograde axonal transport. Autophagosomes are continuously formed at the axon tip while they are rarely formed in dendrites or in the soma [157]. Nascent autophagosomes do not incorporate membranes from the plasma membrane or mitochondria, but rather are formed from specialized ER membrane domains [157]. Proteins are then recruited on autophagosomes in an ordered way, and distal biogenesis possibly facilitates degradation of mitochondria and proteins that reach the tip of the axon by anterograde axonal transport [157]. Constitutive autophagy in neurons is fundamental for neuronal survival, and autophagy dysfunctions have been detected in several neurodegenerative and age-related diseases [158]. Autophagy, as well as other membrane trafficking processes, is regulated by Rab proteins [159]. There are some indications that neurotrophins and, in particular, neurotrophin receptors are involved in autophagy [160,161]. Indeed, it has been demonstrated that p75NTR is required for Purkinje neuron survival in the presence of trophic support, while trophic factor withdrawal induces autophagy [161]. Furthermore, TrkA activation in human glioblastoma cells can induce autophagy [160]. It will be interesting to investigate the possible relationship between Rab proteins and neurotrophin receptors with respect to autophagy.

### 4.6. Rab Proteins and Microtubule Motors

A number of Rab proteins are able to interact directly or through their effector proteins with microtubule motors and are thus good candidates for having a role in axonal transport, whose key players are indeed molecular motors.

For example, Rab6A and Rab6A’ promote microtubule-dependent recycling of Golgi enzymes to the ER starting from the TGN [162]. Constitutively active Rab6A and Rab6A’ mutants are localized to the ER compartment, in contrast to the wildtype proteins, which are mainly present at the Golgi, and reduced levels of Rab6A and Rab6A’ perturb the Golgi apparatus, resulting in aberrant morphology of this compartment and an impairment in Golgi-to ER recycling [162]. Importantly, Rab6A and Rab6A’ interact with dynactin, a complex important for dynein activity, and overexpression of a dynactin subunit efficiently inhibits Golgi-to-ER recycling, strongly suggesting that the function of Rab6A and Rab6A’ is mediated by dynactin [162].

Alternatively, GTP-bound Rab14 directly binds kinesin 16B, and expression of a dominant negative form of Rab14 impairs biosynthetic transport of FGFR from the Golgi to endosomes, thus altering signaling and leading to defects in early development [163]. These data link defects in microtubule-based membrane trafficking to development and could be of great relevance for neurons [163].

Examples of Rab effectors directly interacting with microtubule molecular motors are Rip11/FIP5 and Rab11/FIP3. Rip11/FIP5, a Rab11 effector protein present on peripheral endosomes and fundamental for the slow recycling pathway, interacts with kinesin 2 [164]. Rab11-FIP3, another Rab11 interactor, interacts with dynein light intermediate chain 1 and recruits it on Rab11-positive membranes [165]. This interaction is needed in order to accumulate endosomal-recycling compartment proteins pericentrosomally [165]. Thus, Rab11-FIP3 is important to link Rab11 to dynein in order to transport material to the pericentrosomal recycling endosomal compartment [165].

Investigating the role of Rab proteins interacting with microtubule and actin filament molecular motors either directly or through their effector proteins in neuronal cells will surely lead to the identification of new regulators of axonal transport.

## 5. Neurodegenerative and Age-Related Diseases: Consequences of Alterations of Neurotrophin Trafficking

The axonal transport machinery is essential for neuronal differentiation, survival and plasticity; thus, defects in trafficking and vesicular transport are implicated in many neurodegenerative and age-related diseases [166,167]. Given the important role of neurotrophin signaling, alterations in neurotrophin receptor trafficking are highly detrimental for neurons, which is a key factor for several human diseases.

### 5.1. Alzheimer’s Disease (AD)

AD, a neurodegenerative disorder that is commonly observed in people over 65 years, is characterized by memory loss and cognitive decline. It is also known as senile dementia and is caused by the formation of β-amyloid peptide (senile plaques) in the brain and neurofibrillary tangles containing microtubule-associated Tau protein in the neuronal cell body. β-amyloid peptide is generated by sequential proteolysis by β- and γ-secretase of amyloid precursor protein (APP), a transmembrane protein that is highly expressed in the brain [168]. Alzheimer’s disease is characterized by abnormal accumulation of β-secretase and increased production of β-amyloid peptide, with β-secretase-containing vesicles moving retrogradely in dendrites and in both directions in axons [169,170]. There is a strong link between AD and changes in neurotrophin axonal transport. β-amyloid oligomers impair retrograde trafficking of BDNF/TrkB activated complexes, thus altering synaptic plasticity, which is important for memory and cognition [171,172]. NGF is important to determine Tau protein levels. Indeed, NGF stimulation of PC12 cells causes an increase in Tau protein levels and induces Tau dephosphorylation, while in the absence of NGF, hyperphosphorylation of Tau is observed [173,174]. Furthermore, in the cortex and hippocampus of AD brains, mRNA and protein levels of BDNF and TrkB are significantly reduced [175,176,177]. Anterograde axonal transport of APP is trigged by direct binding of APP to kinesin-1 [178] and it is regulated by Rab3A [106]. In addition, several different Rab proteins are involved in the regulation of APP activity and, therefore, of β-amyloid peptide levels [169]. Rab11 controls endosomal recycling of β-secretase to the plasma membrane and β-amyloid peptide production, which decreases when Rab11 is silenced [179]. Eps15-homology-domain-containing (EHD) 1 and 3 proteins coordinate Rab-mediated endocytic trafficking and are present together with Rab11 on β-secretase-containing vesicles [170]. Expression of dominant negative mutants of EHD1 or of EHD3 drastically reduces retrograde transport of β-secretase-containing vesicles, indicating that Rab11, forming a complex with EHD protein via Rab11FIP2, is a main regulator of trafficking of β-secretase-containing vesicles [170]. In hippocampal neurons and in cholinergic basal forebrain neurons of AD patients, microarray analysis revealed that *rab4*, *rab5* and *rab7* gene expression levels were up-regulated during disease progression, whereas expression of *trk* genes was down-regulated [180,181]. Thus, an increased endocytic flux could be responsible for the decrease in neurotrophin receptor signaling due to premature degradation of signaling complexes, contributing to neurodegenerative progression [180,181]. Anterograde axonal transport of APP is also regulated by presenilins (PS) [182], which are transmembrane proteins that regulate the proteolytic event of*γ*-secretase [183]. Moreover, PS interact with a number of Rab proteins, such as Rab11 and Rab6 [184,185], thus probably acting as a regulator of vesicular trafficking [186]. Indeed, in cells overexpressing PS or in PS-silenced cells, there are abnormal Trk signaling and trafficking, thus reinforcing the idea that PS regulate neurotrophin axonal transport and signaling by acting in concert with a number of Rab proteins [187].

### 5.2. Amyotrophic Lateral Sclerosis (ALS)

Also known as a motor neuron disease (MND), ALS is a fatal progressive neurodegenerative disease caused by loss of motor neurons in the brain and in the spinal cord. In ALS patients, the ability for the brain to initiate and control muscle movement is progressively lost, leading to total paralysis and eventually death. There are several different types of ALS. The main form (90%–95% of ALS cases) is sporadic and it is not due to genetic inheritance. Mutations in at least 15 several genetic loci, including *C9orf72*, *SOD1*, and *ALS2*, cause approximately 30% of familial ALS cases [188]. A transgenic mouse model with human superoxide dismutase 1 (SOD1) mutations shows altered axonal transport [189]. Unlike control mice, SOD1 mutants interact with the dynain-dynactin complex [190]. Additionally, the flavonoid 7,8-dihydroxyflavone (7,8-DHF), a TrkB agonist that induces the same effects of BDNF, has a neuroprotective effect, improving differentiation and survival of motor neurons in a SOD1 mutant mouse model [191]. p75NTR is highly expressed in a mouse model of ALS carrying a SOD1 mutation, indicating a potential role of p75NTR in disease progression and axonal degeneration, and its extracellular domain is cleaved following pro-apoptotic ligand binding [192,193]. In SOD1 mutant mice, there is an accumulation of intracellular APP in abnormally enlarged APP- and Rab7-positive endosomal compartments, indicating an impairment in retrograde vesicle trafficking [194,195]. The correlation between alterations in vesicle trafficking and ALS is corroborated by another mutation found in familial ALS, Alsin2 protein (ALS2), a putative guanine-nucleotide exchange factor for Rab5 and Rac1 [196,197]. The interaction between ALS2 and Rab5 can modulate the signaling of neurotrophins [198]. In fact, ALS2-defective mutant mice showed a strong decrease in endosome trafficking via Rab5 and abnormalities in BDNF endosomal transport [199].

Another cause of familial ALS is a hexanucleotide (GGGGCC) repeat expansion in the intronic region of chromosome 9 open reading frame 72 (C9orf72). C9orf72 is structurally related to DENN (differentially expressed in normal and neoplasia) Rab exchange factor [200]. In neuronal cell lines and primary cortical neurons, C9orf72 colocalizes with Rab1, Rab5, Rab7 and Rab11, and regulates endocytosis and autophagy [201]. Interestingly, depletion of C9orf72 inhibits internalization of TrkB [201]. In motor neurons of ALS patients bearing the C9orf72 mutation, there is an increase in association between C9orf72 and Rab7 and Rab11, suggesting that there is an impairment of endosomal trafficking in these patients [201].

### 5.3. Charcot-Marie-Tooth Disease

Charcot-Marie-Tooth disease (CMT) is the most common inherited neuromuscular disorder, with a prevalence of 1 per 2500 individuals, and more than 40 genetic loci associated with different forms of the disorders have been identified. It is a degenerative nerve disorder that causes muscle weakness and atrophy in the feet, legs, hands and forearms. Muscles fail to receive stimulation from the nerves, and then begin to waste away, leading to atrophy. There are several forms of CMT. Charcot-Marie-Tooth hereditary neuropathy type 2B (CMT2B) is an axonal (non-demyelinating) peripheral neuropathy, clinically characterized by mild sensory loss, normal or near-normal nerve conduction velocities and distal muscle atrophy. This form of the disease is also characterized by a high frequency of foot ulcers and infections that often result in amputations of the toes and of the feet and, thus, is also called ulcero-mutilating neuropathy [202,203,204]. CMT2B is caused by five missense point mutations in the Rab7 protein: L129F, K157N, N161I, N161T and V162M [205,206,207,208]. As retrograde transport of neurotrophin-TrkA and -TrkB receptor-active complexes in signaling endosomes along the axon is under the control of Rab5 and Rab7 proteins [68,130], CMT2B-causing Rab7 mutant proteins could alter neurotrophin trafficking and signaling. In fact, expression of CMT2B-causing Rab7 mutant proteins impairs neurite outgrowth and neuronal differentiation in several different cell lines [153,209]. Furthermore, expression of CMT2B-causing Rab7 mutant proteins alters axonal transport, destabilizing the equilibrium between the rates of anterograde and retrograde transport [210]. Indeed, in rat dorsal root ganglion (DRG) neurons expressing the CMT2B Rab7 mutant proteins, anterograde transport was increased, with vesicles bearing CMT2B Rab7 mutants being faster in the anterograde direction compared to vesicles bearing wildtype Rab7 [210]. Furthermore, expression of CMT2B-causing Rab7 mutant proteins decreased binding of neurotrophins at the cell surface and decreased ERK1/ERK2 activation, thus suggesting premature degradation of TrkA and premature termination of signaling [210].

### 5.4. Huntington’s Disease (HD)

HD is an inherited autosomal-dominant neurodegenerative disorder, characterized by impairment of movement, cognitive decline and emotional problems, with onset in the middle age. HD is characterized by the loss of striatal and cortical neurons and it is caused by a polyglutamine expansion in the Huntingtin (Htt) protein. Htt is a 350 kDa ubiquitously expressed vesicle-associated protein localized in different cellular compartments: the Golgi [211], early endosomes [212] and recycling endosomes [213]. The polyglutamine repeats cause misfolding and aggregation of the Huntingtin protein [214].

There is strong evidence that HD is associated with alterations in axonal transport. In striatal neurons, expression of Htt mutants disrupts fast axonal transport [215]. Htt interacts with microtubule motors via HAP-1 (Htt-associated protein 1), as well as with dynein intermediate chain [216] and kinesin-1[217], thus driving anterograde and retrograde transport. Htt, associated with HAP-1 and the p150^Glued^ subunit of dynactin, increases BDNF vesicular transport, which is decreased in HD [218]. Moreover, phosphorylation of Htt at serine 421 by Akt controls both anterograde and retrograde transport of BDNF in neurons. Phosphorylation induces anterograde transport, recruiting kinesin-1, while dephosphorylation of Htt removes kinesin-1 and favors dynein-mediated retrograde transport [219]. Htt is also a regulator of BDNF-TrkB dendritic retrograde transport in the striatum. In HD, this transport is altered, resulting in neurodegeneration. Indeed, signaling through ERK activation and c-fos induction is decreased [220]. A similar situation is observed upon either silencing of Htt or expression of a mutant form of Htt [220].

Importantly, Rab proteins have a key role in HD. Htt interacts with the optineurin-Rab8 complex, which is involved in post-Golgi trafficking [221]. The Rab8 GTPase regulates trafficking of TGN to the plasma membrane and optineurin is one of its effectors [211]. A mutant form of Htt exhibits reduced interaction with the optineurin-Rab8 complex, causing altered post-Golgi trafficking to lysosomal compartments [211]. In addition, the Htt and HAP40 (Htt-associated protein 40) complex is a Rab5 effector that controls early endosomes motility [212]. In HD, HAP40 is up-regulated, causing a decrease in motility of Rab5-positive endosomes [212]. As Rab5-positive endosomes are involved in retrograde transport of activated neurotrophin/receptor complexes, it is possible that altered Rab5-mediated trafficking of neurotrophins has a relevant role in the development of HD. Moreover, Rab5 overexpression reduces toxicity of the Htt mutant protein, while inhibition of Rab5 increases toxicity via macroautophagy regulation [222].

Rab11, a Rab proteins involved in endosomal recycling, has a prominent role in HD. Indeed, Htt is present in a complex with Rab11 and stimulates Rab11 activation by inducing nucleotide exchange [213,223,224,225]. Consistently, in cell and animal models of HD, Rab11 is less active, thus causing delayed recycling of cargoes from recycling endosomes to the plasma membrane [213,223,224,225]. Furthermore, Rab11-positive vesicles exhibited alterations of transport upon reduction of Htt, of dynein or of kinesin, demonstrating that Htt as well as both motors are required for correct transport of Rab11-positive vesicles [226]. In addition, Rab11, which controls trafficking of the glutamate/cysteine transporter EAAC1 from the recycling endosomes to the plasma membrane, has also a role in oxidative stress, one of the causes of neurodegeneration in HD [227]. Indeed, Rab11 dysfunction caused by mutant Htt impairs EAAC1 delivery to the plasma membrane, resulting in reduced cystein uptake and deficient glutathione synthesis [227]. Furthermore, Rab11 dysfunction impairs glucose uptake in HD, possibly because of altered recycling of glucose transporters to the plasma membrane [228]. Importantly, expression of a constitutively active Rab11 mutant is able to restore endosomal recycling, thus reducing oxidative stress and normalizing glucose uptake [224,227,228]. Moreover, in a Drosophila model of HD, overexpression of Rab11 is able to revert early synaptic dysfunctions, such as the decrease in presynaptic vesicle size, the reduced quantal amplitudes and evoked synaptic transmission, and the alterations of larval crawling [229]. All of these data indicate a central role for Rab11 in HD and suggest that restoring Rab11 activity could be a targeted therapy for HD, as it could revert several dysfunctions.

Alterations of autophagy, a process important to maintain cellular homeostasis in neurons, have been associated with a number of neurodegenerative diseases, including HD [230]. Recently, it has been demonstrated that two distinct populations of Rab7-positive vesicles move retrogradely, and one population is labeled with the LC3 autophagy marker, thus representing autophagosomes [230]. Knockdown of JIP1, a protein that binds to dynein and kinesin-1, does not alter the biogenesis of autophagosomes at the axonal tip, but alters their axonal retrograde motility [230]. Thus, JIP1 is a motor adaptor protein important for retrograde axonal transport of Rab7-positive autophagosomes [230].

### 5.5. Aging

Age-related functional defects such as cognitive decline, sensory perception and motor impairment are characterized by degenerative changes in the central and peripheral nervous systems due to a significant reduction in neuronal plasticity [231,232]. Indeed, while a significant loss of sensory and motor neurons is not detected, with age, neurons show changes in gene expression, morphology and connectivity, leading to degeneration [233]. Degeneration is due, at least in part, to changes in expression of neurotrophin and/or neurotrophin receptors [231,232]. For example, in aged rats, a decrease in all Trk receptors was observed and an attenuation of neurotrophic signals with advancing age has been suggested [231]. Reduced transcription, impaired protein synthesis and processing of BDNF, accompanied by decreased activation of the TrkB receptors, has been detected in aged rats, suggesting that therapeutic strategies could be based on restoring neurotrophin levels and functions [232]. Furthermore, disruption of axonal retrograde transport of neurotrophin might contribute to age-related diseases such as Alzheimer’s disease and Down syndrome, suggesting the involvement of Rab proteins [234]. In fact, the expression profile of cells committed to senescence has been studied in various systems and alterations in Rab protein expression have been detected [235,236,237]. For instance, Rab3a expression decreases during aging and in Alzheimer’s disease [237]. However, an increase in membrane-associated Rab3a has been detected in aging mice [238,239]. Notably, altered lysosomal functionality has been related to aging and age-related diseases, indicating that positive lysosomal modulation, obtained also by increasing Rab function and thus trafficking, could be a strategy to treat age-related protein accumulation disorders [240]. Moreover, a study in C. elegans showed that Rab5 and its interacting protein Rabx-5 are responsible for disorganization of the endosomal compartment that occurs during aging, as Rabx-5 mutants age faster [241]. Furthermore, loss of Rab Escort protein 1 (REP-1) function, which is important for prenylation of Rab proteins, causes age related changes [242]. Interestingly, deficiency of the pro-neurotrophin receptor sortilin prevents age-dependent degeneration of sympathetic neurons in mice and protects mice with lesioned corticospinal neurons from death [243]. Notably, as Rab7b interacts with sortilin and regulates sortilin trafficking, it will be interesting to test the role of Rab7b in neurons, looking in particular at aging [122].

Thus, the data obtained on aged animals indicate that an important factor in aging is represented by decreased neurotrophin and neurotrophin receptor expression levels, accompanied by alterations of Rab protein expression that could modify neurotrophin receptor trafficking.

## 6. Conclusions

The discovery of neurotrophins has opened the doors to a new and unexplored world that is slowly revealing all its complexity, unraveling a complex network of interactions. Neurotrophins and their receptors, through different signaling pathways, participate in many processes leading to neuronal development and functions, such as axonal growth, dendritic branching, synaptic plasticity, cell survival or cell death. Each of these processes consists of several steps, which are not completely known or characterized. Small alterations of neurotrophin trafficking and signaling have devastating consequences on neuronal functionality. The hot spot for excellence is represented by altered axonal transport. Indeed, alterations of axonal transport lead to several neurodegenerative diseases, such as AD, ALS, CMT and HD.

In this review, we have analyzed the involvement of a number of Rab proteins in trafficking and signaling of neurotrophin receptors. Rab proteins have a predominant role in the regulation of vesicular cellular trafficking, through which a great number of processes, such as cell proliferation, cell nutrition, innate immune response, mitosis and apoptosis, are settled. In the case of neurotrophin receptor trafficking and signaling, Rab proteins could represent the “Ariadne’s thread” for a better understanding of the physiological and pathological mechanisms because the neurotrophin-receptor complexes take advantage of vesicular transport regulated by Rab proteins (such as Rab5 and Rab7 in the case of “signaling endosomes” and Rab11 in the case of recycling endosomes) in order for their signaling to reach the target sites.

There is still much to be explored to unravel this tangle of interactions involved in neurotrophin receptor trafficking and signaling where Rab proteins are key players. Clearly, motor and sensory neurons are more vulnerable to small impairment in the vesicular transport than other cell types because they are characterized by very long axons. Moreover, the bottleneck in the study of neurodegenerative disease is represented by the lack of suitable experimental models, due to the large amount of time for the onset of the disease. In fact, these disorders usually show the first signs in the early or, more frequently, late adulthood. The understanding of the precise role of Rab proteins in the regulation of neurotrophin-dependent processes will lead to the discovery of some of the underlying mechanisms of neurodegeneration and will open the way to effective treatments.
